# Identification and molecular analysis of 11 cases of the *PTS* gene variants associated with tetrahydrobiopterin deficiency

**DOI:** 10.3389/fgene.2022.919209

**Published:** 2022-09-12

**Authors:** Lulu Li, Haihe Yang, Jinqi Zhao, Nan Yang, Lifei Gong, Yue Tang, Yuanyuan Kong

**Affiliations:** Department of Newborn Screening Center, Beijing Obstetrics and Gynecology Hospital, Capital Medical University, Beijing Maternal and Child Health Care Hospital, Beijing, China

**Keywords:** HPA, BH4D, PTPS deficiency, PTS, high-throughput sequencing, rare disease

## Abstract

**Background:** Tetrahydrobiopterin deficiency (BH4D) is a rare autosomal recessive amino acid metabolic disease that belongs to a kind of hyperphenylalaninemia (HPA), and 6-pyruvyltetrahydrotrexate synthase (PTPS) deficiency is the most common type of BH4D. This study investigates the clinical and genetic characteristics of 11 PTPS deficiency cases in the Beijing area, identifies the genetic pathogenic factors, and evaluates the value of high-throughput sequencing in the precise diagnosis of PTPS deficiency.

**Methods:** The Beijing Neonatal Disease Screening Center diagnosed patients with HPA. The study used phenylalanine (Phe) in blood, the ratio of Phe to Thr, urotrexate spectrum analysis, erythrocyte dihydrotrexate reductase (DHPR) activity determination, and high-throughput sequencing as methods. Bioinformatics software analyzed the variants’ pathogenicity and used RT-PCR to identify deep intron variants’ pathogenicity.

**Result:** Among 635 cases with HPA, 38 cases were diagnosed with BH4D, of which the incidence in HPA was 5.98%. Nine kinds of PTS gene variants were detected, including seven missense variants, one splicing variant, and one deletion variant. The splicing variant c.84–291A>G had three splicing results *in vivo*: normal length, 79bp pseudoexon insertion, and exon 3 skipping. Bioinformatics and Sanger sequencing were performed to verify the identified variants.

**Conclusion:** High-throughput sequencing is a helpful tool for clinical diagnosis and differential diagnosis of BH4D. This study confirms that c.84–291A>G is the hot spot variant of PTPS deficiency, and it is the first reported variant with a new splicing pattern *in vivo*. A novel deletion variant c.84_163del (p.Lys29Cysfs∗9) was found to enrich the genetic variant spectrum of the disease.

## Introduction

Hyperphenylalaninemia (HPA) is an autosomal recessive metabolic disorder of amino acids. It can be divided into two main categories: phenylalanine hydroxylase (PAH) deficiency and tetrahydrobiopterin deficiency (BH4D) (Blau et al., 2010; van Spronsen et al., 2021). PAH deficiency is due to reduced or deficient activity of phenylalanine hydroxylase (PAH). BH4D is caused by the synthesis of cofactor tetrahydrobiotrexate or by a congenital defect of an enzyme in its metabolic pathway. The two treatments are different (Ye et al., 2013; Singh et al., 2014). There is no clinical manifestation in the initial stage of BH4D, except for the increase in blood phenylalanine (Phe), which means it is easy to be misdiagnosed as the PAH deficiency type and adopt the low Phe diet therapy. Although the blood Phe of the children decreases quickly after treatment, progressive neurological damage symptoms will appear and lead to severe irreversible consequences. Therefore, it is essential to differentiate and give appropriate treatment to HPA children as soon as possible after neonatal disease screening.

BH4D is an autosomal recessive hereditary disease that can be divided into five types (van Spronsen et al., 2021), 6-pyruvyltetrahydrotrexate synthase (PTPS) deficiency, dihydroterine reductase (DHPR) deficiency, guanylate triphosphate cyclization hydrolase (GTPCH) deficiency, trexin-4A-dimethoxylamine dehydrase deficiency (PCD), and sepiapterin reductase (SR) deficiency. They are caused by variants of 6-pyruvyltetrahydrotrexate synthase (*PTS*), dihydroterine reductase (*DHPR*), guanylate cyclization hydrolase I (*GTPCH1*), trexin-4A-dimethoxylamine dehydrase (*PCD*), and sepiapterin reductase (*SR*), respectively (Song et al., 2020), and the lack of any enzyme will lead to the obstruction of BH4 synthesis (Opladen et al., 2012). It is important to emphasize that PTS deficiency accounts for the largest proportion. PTS gene variant makes dihydroneopterin triphosphate unable to produce 6-acetonyl hydropterin, resulting in the increase of neopterin and biopterin synthesis being impaired (Opladen et al., 2012; van Spronsen et al., 2021). BH4D has noticeable regional differences; the prevalence of BH4D was higher in the northern regions (4.1‰) of China than in the central regions (3.4‰) or southern regions (1.6‰) (Ye et al., 2001; Wang et al., 2006; Yuan et al., 2021). More interesting, the total proportion of BH4D cases among HPA cases was 3.9%, and the highest proportion occurred in the southern region (15.1%), while the lower proportion was observed in the northern region (2.0%). (Ye et al., 2001; Wang et al., 2006; Yuan et al., 2021). At present, the differential typing of HPA mainly depends on a variety of comprehensive diagnosis methods such as blood Phe detection, urinary methotrexate spectrum analysis, DHPR activity determination, and BH4 load test (Song et al., 2020). However, the whole differential typing process requires several tests, biochemical detection is influenced by many factors and is not precise sufficiently. Gene testing could reduce false-positive results and facilitate timely diagnostics of HPA (Cao et al., 2014). In addition, it can quickly identify and classify the type of HPA, especially for the differential diagnosis of BH4D, which has been widely used in the clinic.

This study comprehensively analyzed the clinical diagnosis and genetic testing results of 11 children with PTPS deficiency in the Beijing Neonatal Disease Screening Department and explored the genetic etiology of PTPS deficiency and the value of high-throughput sequencing in the early diagnosis of PTPS deficiency.

## Methods and materials

### Family recruitment and ethical sight

In a total of 635 cases of HPA, one patient from another city was hospitalized due to clinical symptoms, and all the other patients, whose heel blood Phe concentration was >2 mg/dl (120umol/L), were collected in Beijing Neonatal Disease Screening Department from 1989 to 2021. We identified 38 individuals with a clinical diagnosis of BH4D, and 11 of them were available with reliable clinical and molecular data. Eleven patients and families with PTPS deficiency were ultimately diagnosed by urinary butterfly spectrum analysis, DHPR activity determination, and gene detection. All the subjects in this study had no family history, and their parents had normal phenotypes and were not consanguineous in marriage. This study was approved by the Ethics Committee of Beijing Obstetrics and Gynecology Hospital affiliated with Capital Medical University, and family members (or guardians) all signed informed consent.

### Urotrexate spectrum analysis and erythrocyte DHRP activity determination

The study collected fresh urine, and vitamin C was immediately added, mixed, and dropped on filter paper to dry naturally. HPLC determined urine neopterin (N) and biopterin (B) to screen for BH4 deficiency. The drops of heel blood sampling were taken and dropped on the filter paper to detect DHPR activity. Meanwhile, each test measured standard control samples, and the percentage (%) of the samples was measured as the regular control activity. Shanghai Xinhua Hospital completed the aforementioned two projects.

### Blood sample collection and DNA extraction

The blood sample was drawn from the affected and normal individuals. Genomic DNA was extracted using the phenol-chloroform method and was quantified using Nanodrop-2000 using standard methods.

### High-throughput sequencing

Genomic library preparation: genomic DNA was randomly interrupted and spliced with primers to prepare the required library; hybridization capture: IDT’s xGen Lockdown system was used to lockdown the probes with the target region libraries of the relevant genes in the samples to enrich the DNA sequences in the exon region; sequencing: Illumina Novaseq high-throughput sequencing was performed on qualified products; data analysis: sequence data were analyzed, filtered, and compared with the human *PTS* (NC_000,011.10) gene reference sequence. This study mainly focused on nonsynonymous, homozygous, or compounded heterozygous variants with a minor allele frequency of 1% (dbSNP142 or ExAC) retained to identify plausible pathogenic variants. The variants were cross-checked in the Human Gene Mutation Database (HGMD; http://www.hgmd.cf.ac.uk/ac/index.php) to know whether that identified variants are novel or already reported.

### Primer designing and mutation confirmation

The *PTS* (NM_00,031.7) gene sequence was obtained from the website (http://genome.ucsc.edu). Primer 5.0 software was used to design the specific PCR primers (Supplementary Table S1). The target region was amplified by polymerase chain reaction (PCR) following the manufacturer’s instructions.

### Bioinformatics analysis

Different bioinformatics software, including sorting intolerant from tolerant (SIFT, http://www.sift.jcvi.org/), Polyphen-2 (http://genetics.bwh.harvard.edu/pph2), Mutation Taster (http://www.mutationtaster.org/), and REVEL (https://sites.google.com/site/revelgenomics/) were used for functional effect prediction**.** Meanwhile, variant frequencies were determined in the 1000 Genomes Project, ExAC (http://exac.broadinstitute.org/), and GnomAD ALL (http://gnomad-sg.org/) database. Finally, the American College of Medical Genetics and Genomics (ACMG) 2015 guidelines were used for the interpretation of variants.

### Validation of the splicing variant

RNA analysis was used to confirm if the intronic variant c.84-291A>G affects RNA splicing. The study extracted 2 ml venous blood from patient-1 and used TRIzol (Invitrogen, Cat No.15596018) reagent to isolate RNA. Reverse transcriptase-PCR (RT-PCR) was performed using oligo dT (Promega, Cat. No. A5001). Nest-PCR was used to amplify the target cDNA fragments, and T-clones (Pmd19-T Vector Cloning Kit, Takara) were used to analyze the sequence of the amplicons.

## Results

### Clinical characteristics

In this study, among 635 cases with HPA, 38 cases were diagnosed with BH4D. BH4D accounted for 5.98% (38/635) of HPA. Due to technical limitations and many families refusing to do gene testing, so many patients are only diagnosed with BH4D, and failed to carry out accurate clinical classification. At the same time, many patients have been lost to follow-up. Thus, 11 of 38 cases in this study have complete clinical molecular data and were diagnosed with PTPS deficiency. Of the 11 cases, four were males and seven were females, including a pair of twins (patient-1 and patient-2). The other 10 were newborn disease screening cases except for patient-10, who was hospitalized due to motor development delay and abnormal muscle tone. Most of the patients have normal skin and hair color, and no symptoms such as eczema, convulsion, dystonia, or other neurological abnormalities (Table 1). Seven cases have continued treatment and regular follow-up, and four cases have stopped treatment.

**TABLE 1:** Clinical and genetic characteristics of patients recruited in this study.

Patient	Gender	Birth weight(g)	Birth height(cm)	Initial Phe level(mg/dl)	Age at first visit	Diagnosis Phe level(mg/dl)	Age at diagnosis of HPA	Age at diagnosis of PTPS	Age at last visit	Clinical characteristics										Genetic characteristics						
										The color of hair	The color of skin	Dystonia	Neurological abnormalities	N (mmol/molCr)	B (mmol/molCr)	B% [B/(N+B)%]	DHPR (%)	BH4 reactivity	Continuous treatment	Gene	Zygote type	Allele origin	Variant location	Nucleotide (amino acid) change	Novel variant
1*	female	2400g	48	5.83	13d	2.4	20d	33d	7m	Normal	Normal	N	N	0.99	0.01	1.38	82.54	Y	Y	PTS	C-het	P	E5	c.286G>A (p.D96N)	N
																						M	I1	c.84-291A>G	N
2*	female	2350g	47	4.86	13d	2.7	20d	33d	7m	Normal	Normal	N	N	1.2	0.03	2.04	103.49	Y	Y		C-het	P	E5	c.286G>A (p.D96N)	N
																						M	I1	c.84-291A>G	N
3	female	2650	49	9.99	16d	7.9	17d	25d	1yr 1m	Normal	Normal	N	N	1.95	0.03	1.4	133.97	Y	Y		C-het	P	E5	c.272A>G (p.K91R)	N
																						M	E5	c.259C>T (p.P87S)	N
4	male	2520	47	11.82	11d	26.8	13d	25d	1yr 10m	Normal	Normal	N	N	1.93	0.07	3.49	106.67	N/A	Y		C-het	P	E2	c.84_163del(p.Lys29Cysfs*9)	Y
																						M	E6	c.317C>T (p.T106M)	N
5	female	3780	50	2.52	12d	11.9	18d	25d	1m	Normal	Normal	N	N	4.09	0.17	3.98	59.68	N/A	N		C-het	P	E5	c.286G>A (p.D96N)	N
																						M	I1	c.84-291A>G	N
6	male	3640	50	8.45	11d	37.1	16d	44d	2yr 11m	Normal	Normal	N	N	20.36	0.88	4.14	31.75	Y	Y		hom	P/M	E5	c.259C>T (p.P87S)	N
7	male	3430	51	3.66	15d	14.2	20d	28d	3yr 6m	Normal	Normal	N	N	3.14	0.21	5.65	54.86	N/A	N		C-het	P	E5	c.286G>A (p.D96N)	N
																						M	I1	c.84-291A>G	N
8	female	3410	50	3.3	14d	11	16d	23d	5yr 5m	Normal	Normal	N	N	3.05	0.01	1.9	76.95	Y	N		C-het	P	I1	c.84-291A>G	N
																						M	E5	c.276T>A(p.N92K)	N
9	male	3760	51	2.86	16d	6.9	18d	30d	5m	Normal	Normal	N	N	4.61	0.28	5.71	87.62	N/A	N		C-het	P	E5	c.259C>T (p.P87S)	N
																						N/A	N/A	N/A	N
10	female	3400	50	N/A	15 yr 4 m	2.97	15 yr 4 m	N/A	17yr 5m	Normal	Normal	Y	Y	N/A	N/A	N/A	N/A	Y	Y		C-het	P	E5	c.276T>A (p.N92K)	N
																						M	E5	c.272A>G (p.K91R)	N
11	female	2520	47	12.45	34d	2.76	37d	107d	3yr 10m	Normal	Normal	N	N	2.22	0.06	2.43	54.99	Y	Y		C-het	P	E3	c.166G>A (p.V56M)	N
																						M	E2	c.155A>G (p.N52S)	N

*, Twins; N, neopterin; B, biopterin; d, day; yr, year; m, month; Y, yes; N, no; N/A, not available; hom, homozygote; C-het, compound heterozygote; P, paternal; M, maternal; E, exon; I, intron.

### Biochemical results

The primary blood Phe concentration range of 11 cases with PTPS deficiency was 2.52–12.45 (mg/dl). The Phe level’s minimum value was 2.4 (mg/dl), and the maximum value was 37.1 (mg/dl) (Table 1). The results of the urine neopterin spectrum showed that neopterin (N) increased significantly in other patients except for patients 1, 2, 3, 4, and 11. Except for patient-6, biopterin (B) decreased significantly in all other cases. Only B% of patient-7 and patient-9 were between 5 and 10%, and B% of other cases were less than 5%. Blood DHPR activity was normal in all patients (Table 1).

### Molecular analysis

#### Variant analysis of *PTS* in PTPS-deficient patients

Among the 11 PTPS-deficient patients, only one variant was detected in patient 9, homozygous variants in patient 6, and compound heterozygous variants in other cases (Table 1). There were nine kinds of variants, including seven missense variants [c.286G>A (p.D96N), c.317C>T (p.T106M), c.259C>T (p.P87S), c.276T>A (p.N92K), c.272A>G (p.K91R), c.166G>A (p.V56M), and c.155A>G (p.N52S)], one splicing variant (c.84-291A>G), and one deletion variant [c.84_163del (p.Lys29Cysfs*9)]. Variants were mainly concentrated in exon 5, accounting for 52.38% (11/21). The splicing variant (c.84-291A>G) accounted for the highest proportion, up to 23.81% (5/21). In all the variants that have been mentioned, only the deletion variant [c.84_163del (p.Lys29Cysfs*9) is the novel variant.

SIFT, Polyphen-2, MutationTaster software, and REVEL were used for joint analysis of the seven missense variants, indicating that the variants were pathogenic (Table 2). Meanwhile, all variant-related diseases were highly consistent with the clinical phenotypes of patients, which was supporting evidence of pathogenicity.

**TABLE 2:** Bioinfomatic analysis of PTS variants.

	ExACALL	1000 g 2015 aug all	Gnom AD ALL	SIFT	Polyphen2	Mutation Taster	REVEL	ACMG
c.286G>A (p.D96N)	-	0.00019968	0.00000398	D (0.002)	D (0.92)	D(1)	D (0.817)	LP
c.317C>T (p.T106M)	0.00007855	-	0.0001	D(0)	D(1)	D(1)	D (0.941)	LP
c.259C>T (p.P87S)	0.00005766	0.00039936	0.00007556	T (0.523)	D (0.579)	D(1)	D (0.501)	LP
c.276T>A (p.N92K)	-	-	-	D (0.003)	D (0.986)	D(1)	D (0.821)	P
c.272A>G (p.K91R)	-	-	-	T (0.255)	B (0.114)	D(1)	D (0.754)	P
c.166G>A (p.V56M)	0.00001647	-	0.00001591	D (0.014)	D (0.852)	D (0.581)	D (0.729)	LP
c.155A>G (p.N52S)	0.0002	-	0.0001	D (0.003)	D (0.969)	D(1)	D (0.801)	LP

N, No; Y, Yes; D, Damaging; T, Tolerated; B, benign; LP, Likely pathogenic; P, Pathogenic.

#### Identification of the intronic variant

**FIGURE 1:**
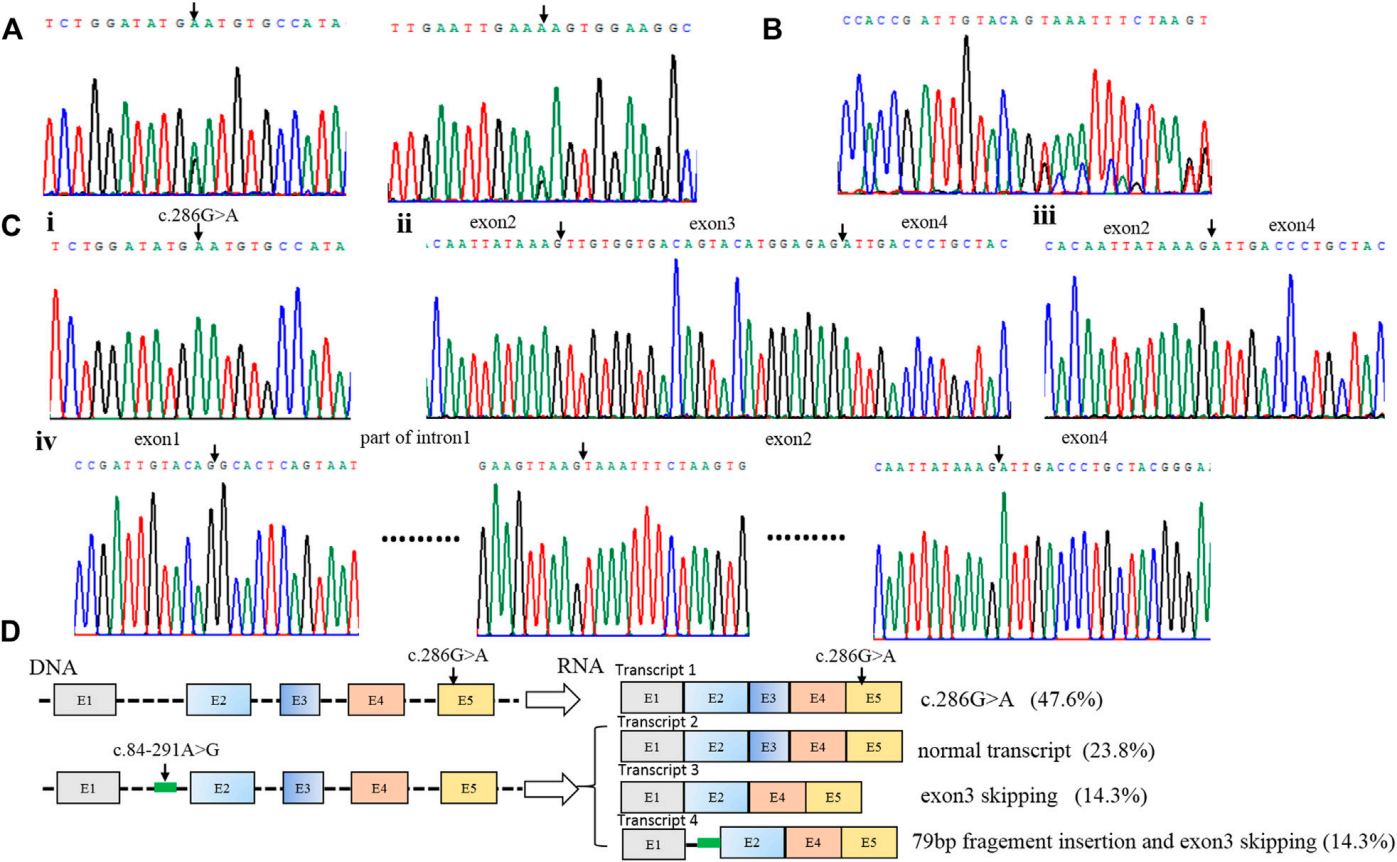
Identification of compound heterozygous variants of *PTS* in patient 1. **(A)** Sequence analysis of genomic DNA with compound heterozygous variants [c.286G>A (p.D96N) and c.84–291A > G] from patient 1. **(B)** RNA analysis of patient 1 by RT-PCR. **(C)** Four forms of the RNA product by T-clone: i. transcript 1 includes a missense variant (c.286G>A); ii. transcript 2 is the normal transcript, including all exons; iii. transcript 3 has exon 3 skipping; iv. transcript 4 includes the 79 bp pseudo-exon and exon 3 skipping. **(D)** Schematic map of the compound heterozygous variants of *PTS* in patient 1. Transcript 1 includes a missense variant (c.286G>A); this variation comes from the paternal allele, and the proportion of this product is 47.6% (10/21); three transcripts are produced by the deep intronic variant c.84–291A>G. Transcript 2 is the normal transcript, and this product accounts for 23.8% (5/21). Transcript 3 has exon 3 skipping, and this product accounts for 14.3% (3/21). Transcript 4 includes the 79 bp pseudo-exon and exon 3 skipping, and it accounts for 14.3% (3/21).

Patient 1 carried the compound heterozygous variant of c.286G>A (P.D96N) and c.84-291A > G (Figure 1A), and RNA was isolated from the blood of the patient, followed by sequencing of RT-PCR products (Figure 1B); RNA analysis showed four forms of transcript product by T-clone (Figures 1C, D). Transcript 1 included a missense variant (c.286G>A); this variation came from the paternal allele (Figure 1Ci), and the proportion of this product was 47.6% (10/21) (Figure 1D).

Three transcripts were produced by the deep intronic variant c.84-291A>G. Transcript 2 was the normal transcript, including all exons (Figure 1Cii), and this product accounted for 23.8% (5/21) (Figure 1D). Transcript 3 had exon 3 skipping (Figure 1Ciii), and this product accounted for 14.3% (3/21) (Figure 1D). Transcript 4 included the 79 bp pseudo-exon and exon 3 skipping (Figure 1Civ), and it accounted for 14.3% (3/21) (Figure 1D).

## Discussion

BH4 is not only the phenylalanine hydroxylase coenzyme but also the coenzyme of tyrosine hydroxylase, tryptophan hydroxylase, and other enzymes. Insufficient or lack of BH4 will affect the activity of PAH and reduce the activity of tyrosine hydroxylase and tryptophan hydroxylase, thus affecting the synthesis of neurotransmitters (such as dopamine and serotonin) in the brain, which results in more severe symptoms of neurological damage and intellectual impairment in children (Leuzzi et al., 2010; Song et al., 2021). Therefore, untreated children with BH4D have more severe clinical symptoms and worse prognosis than those with typical PKU. However, in neonatal BH4D children, there is no clinical manifestation except the increase of blood Phe, and the clinical symptoms often appear only 1–3 months after birth. Due to the completely different treatment methods for BH4D and PAH deficiency, BH4D is easily misdiagnosed as PAH deficiency in the early stages, which delays the treatment opportunity for BH4D. Therefore, the early diagnosis and differential diagnosis of BH4D is vital for clinical treatment (He et al., 2021).

PTPS deficiency is the most common type of BH4D caused by *PTS* gene variant (van Spronsen et al., 2021). In 1992, Thony cloned the cDNA of the *PTS* gene from adult liver cells (Thöny et al., 1992). The human *PTS* gene is located on 11q22.3. It includes six exons and five introns, with a total length of 2 kb and encoding more than 500 nucleotides. Most of the *PTS* gene variants are located in the exon of the coding region or the junction region between exon and intron, and their effect on enzyme activity mainly depends on the form and location of the variant (Thöny et al., 1994; Kluge et al., 1996). Until March 2022, the BH4D-related gene database (http://www.Biopku.org/PNDDB/search-results.asp) has reported 1192 *PTS* gene variants, including missense variants, nonsense variants, splicing variants, deletion, and duplication variants, among which missense variant accounted for the most. The most common variants have been reported as variant c.155A>G (p.N52S), c.259C>T (p.P87S), c.272A>G (p.K91R), and c.286G>A (p.D96N). The variant c.286G>A (p.D96N) is mainly found in the northern population, the variant c.155A>G (p.N52S) occurs predominantly in the southern population; and the variant c.259C>T (p.P87S) is common in the north and south of Chinese population (Ye et al., 2001; Wang et al., 2006; Ye et al., 2013; Li et al., 2018).

In this study, 11 cases with PTPS deficiency were diagnosed, including nine cases with compound heterozygous variants in the *PTS* gene and one patient with homozygous variants, all derived from parents. There is another patient, whose family provided only one *PTS* variant and refused to cooperate with gene testing (Table 1). Twenty-one variants were detected in 11 patients with *PTS*, including nine kinds of variants: c.286G>A (p.D96N), c.317C>T (p.T106M), c.259C>T (p.P87S), c.276T>A (p.N92K), c.272A>G (p.K91R), c.166G>A (p.V56M), c.155A>G (p.N52S), c.84-291A>G, and c.84_163del (p.Lys29Cysfs*9), among which there were seven sorts of missense variants, accounting for 77.78% (7/9). The splicing variant c.84-291A>G accounted for the highest proportion, up to 23.81% (5/21), a hot spot variant of *PTS* in Beijing. Followed by c.286G>A (p.D96N) and c.259C>T (p.P87S), accounting for 19.05% (4/21) and 14.29% (3/21), respectively. The deletion variant c.84_163del (p.Lys29Cysfs*9) has not been reported and it is a novel variant.

The deep intronic variant c.84-291A>G is located in intron 1 of the *PTS* gene. Through RNA cloning, it is found that the variant affects the mRNA splicing process. There are three lengths of splice mRNA: normal length, exon 3 deletions, and 79bp pseudoexon insertion (Figures 1C, D). Due to the variant still having a normal splicing product (23.8%), it can maintain the enzyme activity of partial *PTS* genes, thus, it leads to mild PTPS deficiency, which is consistent with the literature reports (Ye et al., 2001). However, it is the first reported that transcript 4 is the simultaneous existence of 79 bp pseudoexon insertion and exon 3 skipping. In previous reports, only 79 bp pseudoexon was thought to be produced (Liu et al., 2001; Chiu et al., 2012).

We observed that patients 1, 2, 5, 7, and 8 all carry the variant c.84-291A > G. The initial screening blood values of these cases were between 2 and 6 (mg/dl). Patients 5, 7, and 8 stopped drug treatment at the early stage and were under follow-up observation. The phenylalanine level was in the reference range (<2 mg/dl), consistent with our expectation. In addition, we found that the diagnosis phenylalanine blood values of cases-5, -7, and -8 were 11.9, 14.2, and 11, which we suspect may be related to increased protein compliance or temporary impairment of liver enzyme activity after adequate lactation. Patient 1 and patient 2 are twins who are still under drug treatment. We explained the disease and genes in detail to the patients’ parents and recommended a drug withdrawal observation treatment scheme. However, the parents were very cautious and afraid of adverse consequences caused by drug withdrawal, so they insisted on continuing to take drug treatments.

The variant c.286G>A (p.D96N) means the 286th base from G to A, resulting in the conversion of aspartic acid at 96th to asparagine. It has been reported that this variant leads to the *PTS* enzyme activity of only 10% (Imamura et al., 1999). Patients 1, 2, 5, and 7 are c.286G>A (p.D96N) and c.84-291A > G compound heterozygous, and they are all mild PTPS deficiency.

The variant c.259C>T (p.P87S) means the 259th base from C to T, resulting in the change of 87th proline to serine. This variant resulted in an almost complete loss of enzyme activity (Dudesek et al., 2001; Wang et al., 2006). Patient 3 was a compound heterozygous patient with c.272A>G (p.K91R) and c.259C > T (p.P87S), and the initial screening blood value was 9.99 (mg/dl). Patient-6 was a homozygous patient with c.259C>T (p.P87S), and the initial screening blood value was 8.45 (mg/dl). Both patients were severe PTPS deficiency. In particular, patient-6, whose diagnosis Phe blood value was up to 37.1 (mg/dl), the homozygous variant of P87S that resulted in significant impairment of enzyme activity. Patient-9 only found a variation of c.259C>T (p.P87S) but the initial screening value was low and the disease was mild. We suspect that patient-9 may carry a mild variant. However, the family refused to continue genetic testing, so we have no way to confirm another variant.

So far, some mutants [c.155A>G (p.N52S), c.166G>A (p.V56M), c.272A>G (p.K91R), c.276T>A (p.N92K), and c.317C>T (p.T106M)] have no experimental information on protein stability, or activity is available (Thöny and Blau, 1997). Through literature reports and case summaries, it is suggested that variants c.155A>G (p.N52S) and c.317C>T (p.T106M) may lead to severe clinical manifestations (Liu et al., 2001) and variant [c.166G>A (p.V56M), c.272A>G (p.K91R) and c.276T>A (p.N92K)] may be associated with a mild clinical phenotype (Liu et al., 2001; Manti et al., 2020).

The routine diagnostic process of BH4D is as follows: neonatal screening, recall of suspected positive patients for blood Phe, urine dimethylamine analysis, DHPR activity, and other comprehensive diagnostic analysis methods (Song et al., 2020). In this study, the initial screening blood Phe concentration of all the cases was 2.52–12.45 (mg/dl), after sufficient lactation, the blood value changed greatly. The range was 2.4–37.1 (mg/dl), which could be diagnosed as HPA, but it was challenging to make a differential diagnosis for the classification. Urotrexate spectrum analysis was completed in Shanghai Xinhua Hospital. The experiment involved the storage and transportation of urine samples. The N of patients 1, 2, 3, 4, and 11 did not increase significantly, and patient-6’s B was 0.88, which did not decrease significantly. We considered that this was related to various factors such as urine sample contamination, preservative failure, and sunlight exposure. Compared with fresh urine samples, the storage and transportation of blood plaques were relatively stable, and the activity of blood DHPR was regular in all cases.

PTPS deficiency can be easily diagnosed when B% is less than 5%. However, if the B% is between 5 and 10% (such as in patients 7 and 9), this situation needs to be comprehensively analyzed with other results (Bao et al., 2019; Himmelreich et al., 2021). Therefore, the whole diagnostic process takes a long time. In particular, we found patient-11’s situation that the longest time from the initial diagnosis of HPA to the diagnosis of PTPS deficiency was 73 days, due to problems in the preservation or transportation of samples. Therefore, the diagnosis of BH4 was delayed.

Based on the limitations and long timeliness of the aforementioned methods, gene testing technology has been widely used, especially with the mature high-throughput sequencing technology and rapid development, which can realize the accurate capture of target genes and the comprehensive analysis of high genetic heterogeneity diseases (Li et al., 2020). It has lower operation costs, high-throughput, and fast operation and has reliable and stable results. Meanwhile, it has been successfully applied to the diagnosis and differential diagnosis of BH4D (Liu et al., 2016; Manti et al., 2020). More importantly, high-throughput sequencing can provide genetic counseling for families.

## Conclusion

In this study, we analyzed the clinical situation, differential diagnostic criteria, and gene variants of 11 cases of PTPS deficiency in detail. The hot spot variant of PTPS deficiency in the Beijing area and a new variant of the *PTS* gene were found, which enriched the variant spectrum of the *PTS* gene, and revealed three splicing results of c.84–291A > G *in vivo*. At the same time, it suggested that clinicians should pay more attention to genetic testing, use high-throughput sequencing technology to identify the etiology quickly, and improve disease diagnosis efficiency so that PTPS deficiency patients can be diagnosed early and treated timely and effectively. What’s more, effective genetic counseling and prenatal diagnosis can be provided to the patient’s family.

## Data Availability

The original contributions presented in the study are included in the article/Supplementary Material; further inquiries can be directed to the corresponding author.
