# Supplementary material for: Identification and molecular analysis of 11 cases of the PTS gene variants associated with tetrahydrobiopterin deficiency
Source: Front Genet. 2022 Sep 12;13:919209. doi: 10.3389/fgene.2022.919209 (PMC9536429; doi:10.3389/fgene.2022.919209)
Supplement: Supplementary file 1 [file Table1.docx]

**Supplementary Table 1. Primers used in this study.**

| **Primer name** | **Sequence (5' → 3')** | **T_m_ (°C)** | **Size (bp)** |
| --- | --- | --- | --- |
| *PAH* – F1 | TGTAAAGTTGCCTTGTAAGACTCAA | 60 | 342 |
| *PAH* – R1 | GAAATTCTAGTTTCGAAAGATTTCA |  |  |
| *PAH* – F2 | TGCTAAATAGGAGACTTACCAGGTC | 60 | 311 |
| *PAH* – R2 | ACGTCACAAACCATGGCATA |  |  |
| *PAH* – F3 | AGGTGGGTGGCACTGTAT | 60 | 393 |
| *PAH* – R3 | TCCAAGGGGATCAAAATC |  |  |
| *PAH* – F4 | TATTCAGTAACAAGGATTC | 60 | 498 |
| *PAH* – R4 | CAGTCGTGCTATAAGAAA |  |  |
